# Early Stone Manipulation in Urinary Tract Infection Associated with Obstructing Nephrolithiasis

**DOI:** 10.1155/2018/2303492

**Published:** 2018-11-25

**Authors:** Megan L. Swonke, Ali M. Mahmoud, Elias J. Farran, Tamer J. Dafashy, Preston S. Kerr, Christopher D. Kosarek, Joseph Sonstein

**Affiliations:** Division of Urology, Department of Surgery, University of Texas Medical Branch, Galveston, Texas, USA

## Abstract

A urinary tract infection (UTI) and sepsis secondary to an obstructing stone are one of the few true urological emergencies. The accepted management of infected ureteral stones includes emergent decompression of the collecting system as well as antibiotic therapy. Despite this, no consensus guidelines clarify the optimal time to undergo definitive stone management following decompression. Historically, our institution has performed ureteroscopy with laser lithotripsy (URS-LL) treatment at least 1 to 2 weeks after decompression to allow for clinical improvement and completion of an antibiotic course. In this case series, we retrospectively review four cases in which patients had a documented UTI secondary to an obstructive ureteral stone. The patients underwent urgent decompression and, based on labs and clinical improvement, were subsequently treated with URS-LL. The presented patients received URS-LL within 5 days of decompression and antibiotics. The patients had no sepsis related postoperative complications from the accelerated course of treatment, resulting in discharge within 2 days following URS-LL. We provide a detailed examination of each patient presentation to describe our institution's experience with treating infected kidney stones within days of urgent decompression in order to question the previous standard of treating an infected kidney stone with a more delayed intervention.

## 1. Introduction

Approximately 1 out of every 11 people in the United States reports a history of kidney stones, marking a significant increase in stone disease over the prior 15 years [1, 2]. Treatment of nephrolithiasis over the past decades has shifted away from inpatient settings and open procedures to outpatient environments and less invasive options, such as endoscopy [3]. With increasing prevalence, urolithiasis is estimated to pose a $1.24 billion economic burden per year by 2030 [4].

It is understood that not all patients with stone disease require urgent intervention [5]; however, patients presenting with a urinary tract infection (UTI) in the setting of ureteral stone obstruction require urgent surgical decompression in order to avoid serious complication, including mortality [6]. Current American Urological Associated (AUA) and European Association of Urology (EAU) guidelines suggest that active UTI with stone obstruction be drained with a nephrostomy tube or ureteral stent before treatment of stone in order to allow for the penetration of antibiotics and drainage of infected urine before definitive treatment of the stone [7, 8]. While the literature discusses different methods of decompression of the collecting system [9] as well as factors that influence the timing of initial intervention for decompression [10], there are no evidence-based guidelines to suggest when a complicated stone should be treated. At our institution, the typical standard has been to wait 1-2 weeks after surgical decompression and antibiotics to treat an infected kidney stone. In this study, we review four cases in which patients with UTI in the setting of obstructing stones receive definitive treatment within days of administering antibiotics and decompression with stenting.

## 2. Materials and Methods

We retrospectively reviewed four cases in which patients had a UTI with concomitant upper tract obstruction secondary to urolithiasis. In each patient, the diagnosis was confirmed by noncontrast computed tomography (CT) scan of the abdomen and pelvis. Per established guidelines [7], emergent stenting was performed, followed by various IV antibiotic treatments. Based on labs and clinical improvement, patients were treated with ureteroscopy with laser lithotripsy (URS-LL). The timing of URS-LL, the length of hospital stay, and postoperative complications were documented. All cases were performed between February 2015 and October 2017.

## 3. Case Presentations

### 3.1. Case 1

A 62-year-old female with a past medical history of hypertension presented to the emergency department with worsening left flank pain associated with nausea and subjective fevers. Physical examination findings included left lower quadrant tenderness and left sided costovertebral angle tenderness. The patient had no history of nephrolithiasis.

The patient was afebrile, but her initial vital signs were significant for hypertension (182/95)/MAP (124), tachycardia (109 beats per minute), and tachypnea (20 breaths per minute). The patient's complete blood count (CBC) and serum chemistry panel showed a leukocytosis of 16,500/*μ*L and a creatinine of 1.36 mg/dL. Urinalysis found 44 white blood cells (WBCs) and 16 red blood cells (RBCs) per high-power field (HPF), with positive leukocyte esterase (500/*μ*L), negative nitrites, and no bacteria, and initial urine culture was contaminated. Initial CT scan showed obstructing left sided stones (1 cm calcified stone within the proximal left ureter and a 2.2 cm stone below the ureteropelvic junction) causing hydronephrosis.

The patient was then started on levofloxacin at the time of admission and subsequently underwent a left ureteral stent placement. During stent placement, purulent discharge was noted after cannulation with a guidewire. Postoperative course was unremarkable. The urine culture showed no growth or presence of bacteria after 24 hours. Considering the patient was afebrile, hemodynamically stable, and labs were within normal limits, the patient underwent URS-LL one day following stent placement. The duration of the procedure was 94 minutes. An access sheath was placed and a flexible ureteroscope was advanced into the kidney. A single stone was encountered in the lower pole of the kidney and was fragmented to dust then extracted with a zero-tip basket. Following removal of the stones, the patient clinically improved and was discharged the next day with a stent in place.

### 3.2. Case 2

A 49-year-old female with a past medical history of a solitary right kidney presented with right lower quadrant pain associated with nausea, vomiting, decreased urination, and dysuria. Physical exam findings included right lower quadrant tenderness and right costovertebral angle tenderness. The patient had no history of stones.

**Figure 1:**
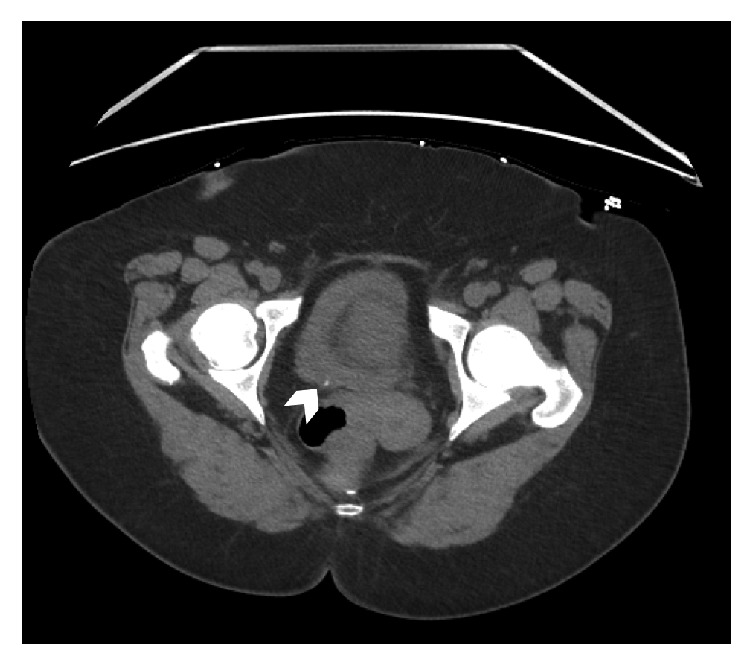
Axial CT scan of the abdomen and pelvis without contrast revealed an atrophic left kidney and a 4 mm stone (arrowhead) in the right ureterovesical junction causing mild hydroureteronephrosis with findings suggestive of pyelonephritis.

During the initial evaluation, the patient's vital signs were significant for hypotension (77/46), tachycardia (117 beats per min), tachypnea (22 breaths per min). She was afebrile; however, had leukocytosis of 24,190/*μ*L, creatinine of 3.90 mg/dL, and lactic acid of 7.49 mg/dL. There was concern for sepsis due to a sequential (sepsis related) organ failure assessment (SOFA) score >2 [11]. Urinalysis found 30 WBCs and 6 RBCs per HPF, positive leukocyte esterase, and negative nitrites. Urine culture grew* Klebsiella pneumoniae*. Initial CT scan showed an atrophic left kidney and a 4 mm stone in the right ureterovesical junction causing mild hydroureteronephrosis with findings suggestive of pyelonephritis (Figure 1).

The patient underwent an emergent cystoscopy with right ureteral stent placement and was started on piperacillin-tazobactam. Intraoperatively, purulent discharge emitted from the right ureteral orifice after cannulation with a guidewire. Her postoperative course was complicated by persistent hypotension requiring vasopressors. Blood cultures revealed Klebsiella and antibiotics were tailored accordingly.

Three days following decompression and continued IV antibiotics, repeat blood and urine cultures were negative for bacteria. On the fifth day, with no obvious signs or symptoms of persistent infection, the patient underwent a URS-LL and right ureteral stent placement with a string. A rigid ureteroscope was introduced into the ureter and a stone was encountered in the distal ureter. Laser lithotripsy was performed, and the stone was obliterated into small fragments which were extracted with a wire basket. Laser lithotripsy was performed instead of a simple extraction to safely extract the stone without causing damage to the ureter. Subsequently, an access sheath was then placed and a flexible ureteroscope was advanced into the kidney. Randall plaques were encountered in the upper pole and were removed using laser lithotripsy. The duration of the procedure was 108 minutes. Following URS-LL, the patient recovered without complications and was discharged on the same day with a completion course of PO antibiotics. The patient was at high risk to be lost to follow up, so was instructed to pull the string to remove the stent on postoperative day 3. Given what was essentially a solitary kidney and the patient's social circumstances, the risk of a retained ureteral stent in this setting outweighed the risks of premature stent removal; thus the stent was left attached to the string to facilitate self-removal of the stent.

### 3.3. Case 3

**Figure 2:**
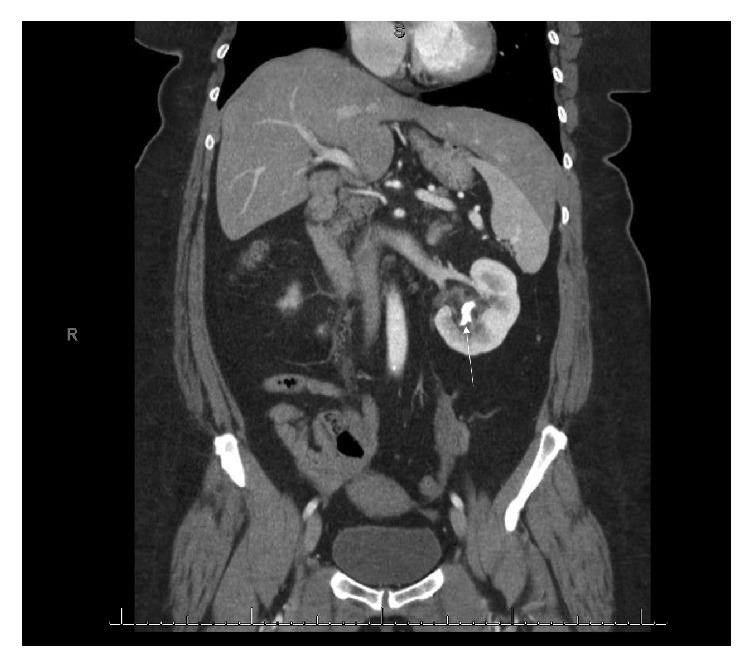
Coronal CT scan of the abdomen and pelvis with contrast revealed a 6 mm obstructing right proximal ureteral stone (not shown in image) causing mild hydronephrosis and a 2 cm left inferior pole staghorn calculus causing calyceal dilatation (arrow).

A 59-year-old female with no significant past medical history presented to the emergency department with acute onset right flank pain radiating to the right lower quadrant. The pain was described as stabbing and constant and associated with nausea and vomiting. She had no prior history of nephrolithiasis. The patient's CBC and serum chemistry were within normal limits. Urinalysis was positive for nitrite, leukocyte esterase (500/*μ*L), 27 WBC per HPF, 23 RBC per HPF, and moderate bacteria. Urine culture revealed the presence of >100,000 CFU/mL* Escherichia coli*. Initial CT scan showed a 6 mm obstructing right proximal ureteral stone causing mild hydronephrosis and a 2 cm left inferior pole partial staghorn calculus causing calyceal dilatation (Figure 2).

The patient was then started on IV levofloxacin and underwent bilateral ureteral stent placement the following day without complication. Urine culture collected during stent placement showed resolution of bacteriuria. Five days after stent placement and continued antibiotic therapy, the patient underwent bilateral URS-LL, complicated by left ureteral perforation upon guidewire placement. The perforation occurred due to a technical error where the stiff end of the PTFE wire was advanced up the ureter and was seen perforating the ureter on fluoroscopy. The wire was removed and then advanced correctly. An access sheath was placed past the perforation and a flexible ureteroscope was advanced. One stone was encountered in the left inferior pole of the kidney and another was encountered in the right proximal ureter. They were both fragmented then extracted with a zero-tip basket. The duration of the procedure was 60 minutes. The patient was discharged after two days of hospitalization. The stents were removed in clinic three weeks postoperatively without complication.

### 3.4. Case 4

**Figure 3:**
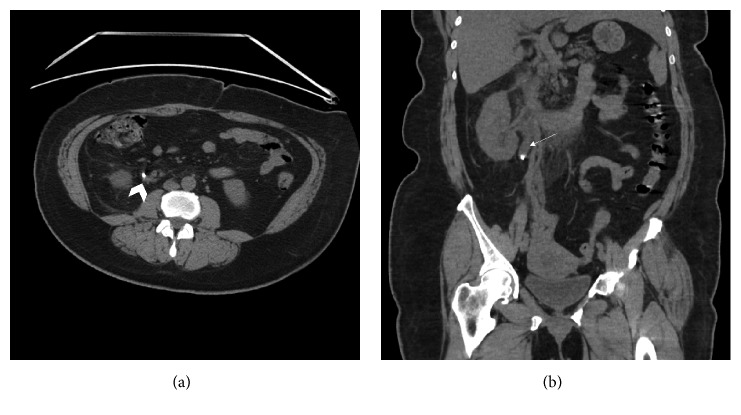
Axial (a) and coronal (b) CT scans of the abdomen and pelvis without contrast revealed an 8 mm right obstructive proximal ureteral stone ((a) arrowhead) causing hydroureter and hydronephrosis ((b) arrow).

A 62-year-old female with a past medical history of hypertension, poorly controlled diabetes, and nephrolithiasis presented to our hospital's emergency department three days after receiving a diagnosis of UTI with multiple nonobstructive renal stones. The patient was found to have right sided abdominal pain radiating to her right flank with fevers, nausea and vomiting, and endorsed dysuria. CBC and serum chemistry panel showed a leukocytosis of 13,740/*μ*L, and creatinine of 1.06 mg/dL. Urinalysis was positive for ketones, proteinuria, urobilin, nitrite, leukocyte esterase, 146 RBC per HPF, over 182 WBC per HPF, and many bacteria. Urine culture was positive for* Escherichia coli*. CT scan performed at our institution showed an 8 mm right obstructive proximal ureteral stone as well as bilateral nonobstructive stones (Figure 3).

The patient was given ceftriaxone and underwent right ureteral stent placement. The patient tolerated the procedure well. Intraoperative urine culture was positive for* E. coli* and she was started on piperacillin-tazobactam. Four days after decompression, she underwent uncomplicated URS-LL. A rigid cystoscope was introduced into the bladder and a flexible grasper was used to grasp and remove the retained ureteral stent. A ureteral access sheath was placed over a guidewire before a flexible ureteroscope was introduced into the ureter through the access sheath. An 8 mm stone was encountered in an interpolar calyx. Laser lithotripsy was performed to obliterate the stone into small fragments which were then extracted with a wire basket. No stent was placed after procedure. The duration of the procedure was 43 minutes. Postoperative course was uneventful, and the patient was discharged later that day.

## 4. Discussion

UTI with concomitant obstructive nephrolithiasis is considered a urologic emergency due to the risk of sepsis, renal loss, and even death. It is well understood that the accepted management of infected ureteral stones entails emergent decompression of the collecting system and antibiotics [7]. Borofsky et al. [5] attested the mortality rate to be higher in patients who did not receive surgical decompression compared to those who received intervention. Neither percutaneous nephrostomy (PCN) nor ureteral stent proved to be a superior draining technique in regard to clinical outcomes [6]. Although controversial, both are currently accepted as the standard of care [6, 9, 10, 12]. However, the optimal time to treat these stones following decompression and empiric antibiotic treatment remains unclear.

Current literature highlights the risk of sepsis following manipulation of ureteral stones in the background of infection [13]. The AUA encourages physicians to delay stone treatment, but does not provide an optimal time to treat the infected stones, only concluding that stone removal should be delayed until infection has resolved and the patient has been treated with an appropriate course of antibiotic therapy [7]. The EAU also reiterates that manipulation should be postponed until the infection has cleared, and the antibiotic course has been completed [8].

Only one published randomized control trial involving 107 patients has explored immediate ureteroscopic management in the setting of sepsis versus PCN for decompression [14]. The trial concluded the length of hospital stay to be longer in the emergent retrograde ureteroscopic management group compared to the PCN group [14]. In addition, the trial concluded a higher analgesic requirement in the emergent retrograde ureteroscopic management group compared to the PCN group [14]. Although patients in the emergent retrograde ureteroscopic management group had a significantly higher incidence of fever, the trial did not find a statistical difference in the groups in regard to level of leukocytosis, time to normalization of WBC (days), time to normalization of body temperature (days), C-reactive protein (mg/dl), C-reactive protein reduction over 25%(days), procalcitonin (ng/ml), and complications rate [14]. The trial advocated for immediate URS-LL when given antibiotics to adequately treat obstructive pyelonephritis [14]. These findings provide additional support for our results, and suggest with proper clinical judgement, URS-LL can be performed within days of system decompression without risk of harm to patients.

Our series presents four patients with UTI and obstructive urolithiasis in which URS-LL was successfully performed within days of antibiotic administration and decompression (Table 1). The common denominator for these four patients who received early treatment is that they were identified as patients who would be at high risk for being lost to follow up with a retained stent based on their lack of access to healthcare resources. This risk of retained stent with its potential complications was considered in the decision making as to whether to manage these stones during their initial admission for infected stone. There is no current guideline regarding the appropriate time for stone treatment after emergent stent placement in the context of an infected stone. All patients were appropriately counseled regarding the inherent risks and potential complications of the procedure and given a thorough informed consent regarding surgery. The patients understood the risks and were amenable to this treatment.

The patients in all four cases presented with symptoms of a UTI secondary to urolithiasis as confirmed by CT scan. Additionally, the patient in Case 2 presented with sepsis consistent with the Third International Consensus Definitions for Sepsis and Septic Shock. Her SOFA score was >2, including a creatinine level >3.5 mg/dL and hypotension requiring the administration of a vasopressor [11]. In Case 2, the patient's blood cultures revealed Klebsiella bacteremia, and in Cases 3 and 4 the patient's urine cultures were positive for* E. coli*. However, the patient in Case 1 had negative cultures at the time of presentation, potentially due to immediate antibiotic dosing at admission. We recognize that in this setting cultures may not always be positive for bacteria; a negative culture does not necessarily exclude infection, particularly a midstream urine catch, or if antibiotics are administered prior to culture causing sterilization within a few hours [5, 15–17]. Empiric antibiotic therapy consisted of broad-spectrum antibiotics, but was adjusted according to cultures [18]. The urine culture in Case 1 was contaminated; therefore, we were unable to tailor our antibiotic choice and levofloxacin was continued based on clinical response. URS-LL was performed when the patient exhibited improvement with decompression and antibiotics. This occurred within one day of decompression.

The highest concern of manipulating an infected stone is the risk of sepsis postoperatively. The four patients had no signs of sepsis postoperatively and were all candidates for discharge on the same day of the procedure with continued antibiotics. Case 3 was kept for an additional two days for pain management. In addition, this patient experienced a complication of wire perforation during initial safety wire placement. We consider this due to technical error, rather than a complication related to her infection.

Our case series has demonstrated successful URS-LL of obstructing stones in the setting of UTIs within days of stenting, several days shorter than our typical institutional standard. If similar outcomes can be replicated in larger prospective randomized controlled studies, treatment standards may clarify a shorter period before definitive surgical intervention in an attempt to optimize patient care and reduce costs.

## 5. Conclusion

Upon reviewing these four cases at our institution, we did not observe any significant adverse outcomes related to sepsis in treating patients within 5 days of decompression when compared to the more conservative approach of withholding treatment for 1 to 2 weeks. It is important to note our case series is limited by the small number of cases reviewed and further studies are needed to clarify the hypothesis generated and the appropriate guidelines regarding the timeline of treatment. Physician awareness should be increased on the feasibility of performing accelerated stone intervention in this setting to optimize patient care.

## Figures and Tables

**Table 1:** Comparing *Cases 1, 2, 3, and 4*.

**Cases**	**WBC**	**Creatinine**	**UA: ** **WBC (per hfu), leukocyte esterase, nitrites**	**Urine Culture**	**Obstructing Stone size**	**Days before URS-LL after decompression**	**Complications after URS-LL**	**Days before discharge after URS-LL**
*Case 1*	16,500/*μ*L	1.36 *μ*mol/L	44, +, -	Contaminated	1 cm and 2.2 cm	1	None	1
*Case 2*	24,190/*μ*L	3.90 *μ*mol/L	30, +, -	*Klebsiella pneumoniae*	4 mm	5	None	0 (same day)
*Case 3*	5,700/*μ*L	0.73 *μ*mol/L	27, +, +	*Escherichia coli*	6 mm (right) and 2 cm (left)	5	Left ureteral perforation	2
*Case 4*	13,740/*μ*L	1.06 *μ*mol/L	182, +, +	*Escherichia coli*	8 mm	4	None	0 (same day)
